# Transcriptome-Wide Mapping of Pea Seed Ageing Reveals a Pivotal Role for Genes Related to Oxidative Stress and Programmed Cell Death

**DOI:** 10.1371/journal.pone.0078471

**Published:** 2013-10-29

**Authors:** Hongying Chen, Daniel Osuna, Louise Colville, Oscar Lorenzo, Kai Graeber, Helge Küster, Gerhard Leubner-Metzger, Ilse Kranner

**Affiliations:** 1 Plant Germplasm and Genomics Center, Germplasm Bank of Wild Species, Kunming Institute of Botany, Chinese Academy of Sciences, Kunming, People's Republic of China; 2 Seed Conservation Department, Royal Botanic Gardens, Kew, Ardingly, West Sussex, United Kingdom; 3 Departamento de Fisiología Vegetal, Centro Hispano-Luso de Investigaciones Agrarias (CIALE), Facultad de Biología. Universidad de Salamanca, Salamanca, Spain; 4 Institute for Biology II, Botany/Plant Physiology, Faculty of Biology, Albert-Ludwigs-University Freiburg, Freiburg, Germany; 5 Institute for Plant Genetics, Unit IV – Plant Genomics, Leibniz Universität Hannover, Hannover, Germany; Queen's University Belfast, United Kingdom

## Abstract

Understanding of seed ageing, which leads to viability loss during storage, is vital for *ex situ* plant conservation and agriculture alike. Yet the potential for regulation at the transcriptional level has not been fully investigated. Here, we studied the relationship between seed viability, gene expression and glutathione redox status during artificial ageing of pea (*Pisum sativum*) seeds. Transcriptome-wide analysis using microarrays was complemented with qRT-PCR analysis of selected genes and a multilevel analysis of the antioxidant glutathione. Partial degradation of DNA and RNA occurred from the onset of artificial ageing at 60% RH and 50°C, and transcriptome profiling showed that the expression of genes associated with programmed cell death, oxidative stress and protein ubiquitination were altered prior to any sign of viability loss. After 25 days of ageing viability started to decline in conjunction with progressively oxidising cellular conditions, as indicated by a shift of the glutathione redox state towards more positive values (>−190 mV). The unravelling of the molecular basis of seed ageing revealed that transcriptome reprogramming is a key component of the ageing process, which influences the progression of programmed cell death and decline in antioxidant capacity that ultimately lead to seed viability loss.

## Introduction

The majority of seeds are orthodox (desiccation tolerant), and undergo dehydration during maturation prior to shedding. In the dry state metabolic activity is minimal, which limits the formation of reactive oxygen species (ROS) that cause oxidative damage associated with ageing, and enables seeds to survive for long periods. However, even in the dry state ROS formation as a result of auto-oxidative reactions leads to a gradual accumulation of oxidative damage to proteins, lipids and nucleic acids, and this eventually leads to viability loss [1], [2], [3], [4]. The molecular basis of ageing has been more widely studied in animals than plants, revealing that ageing is orchestrated by signalling pathways and transcription factors [5]. However, the large-scale transcriptional changes related to seed ageing have not been investigated.

Seeds contain large amounts of stored mRNA, which in the case of *Arabidopsis* seeds represent more than half of all genes [6]. The stored mRNAs are translated into proteins during the early stages of imbibition [7]. Transcription has been reported to occur in dry seeds [8], [9], [10], potentially facilitated by the existence of localised regions of higher water content. Studies of dormancy alleviation during dry after-ripening showed that whilst some transcripts increased in abundance, most declined [11]. This may reflect chemical reactions that are occurring in the dry seed rather than changes in gene expression. For example, nucleic acid oxidation occurs in seeds with moisture content (MC) as low as 4%, and RNA is more prone to oxidation than DNA in part due to its single stranded structure. mRNA was found to be more sensitive to oxidation than total RNA, and oxidation was a targeted process, which would provide a means of modulating cell signaling during the early stages of imbibition [11], [12].

Seed ageing is often studied using artificial ageing or controlled deterioration at higher relative humidity (RH) and temperature to speed up the natural ageing process, so that seeds lose viability within days or weeks rather than decades [13]. In this study the transcriptional changes during the early stages of artificial ageing of garden pea (*Pisum sativum*) seeds at 50°C and 60% RH were investigated using microarrays. This follows on from previous work on nucleic acid integrity [14], redox state [4] and volatile production [15] during the later stages of ageing when seed viability starts to decline. To address the problem of nucleic acid degradation during ageing, which accompanies viability loss [14], RNA integrity was assessed in incrementally deteriorated seed lots, and microarray analyses were conducted only with RNA of satisfactory quality from viable seeds at the initial stages of ageing (up to 15 d). Quantitative real-time (qRT)-PCR analysis was conducted to monitor the expression of selected genes during the entire ageing time course (up to 55 d). In addition, glutathione and other low molecular weight thiol-disulphide redox couples were measured to determine whether the cellular redox state could be related to transcriptional modulation during ageing. This work shows that transcriptional changes during ageing were accompanied by a progressive shift towards a more oxidising cellular environment and nucleic acid degradation, which occurred prior to any loss of seed viability. We propose that seed ageing is not only subject to chemical modification of cellular components but also to genetic control, and that programmed cell death (PCD) is initiated from the onset of ageing, which leads to viability loss.

## Materials and Methods

### Seed material, ageing and germination treatments

Seeds of garden pea (*Pisum sativum* L. cv Alaska Early; Abundant Life Seed Foundation, http://www.abundantlifeseeds.com) were equilibrated at 20°C and 60% RH prior to ageing at 50°C and viability was assessed by germination testing [4]. For molecular analyses, 3–5 biological replicates of 20 pea seeds each were taken at each ageing interval, immediately frozen in liquid nitrogen, freeze dried and ground to a fine powder in a liquid nitrogen-cooled Teflon capsule using a laboratory ball mill (B. Braun Biotech International, http://www.bbraunbiotech.com). The powder was stored at −70°C in humidity-proof vials.

### RNA and DNA extraction and assessment of integrity

RNA was isolated from 30 – 50 mg ground seed powder [16]. RNA quantity and integrity were tested using an Agilent 2100 Bioanalyzer (Agilent Technologies, http://www.agilent.com) together with an Agilent RNA 6000 Nano Labchip kit [17].

Genomic DNA was extracted from approximately 50 mg freeze-dried seed powder [14]. DNA quality and quantity were assessed spectrophotometrically (Jasco, http://jasco.co.uk) at 260 nm and 280 nm. Ten µg of DNA were separated on 1.5% agarose gel, and fragments visualized using a UV transilluminator (Syngene, http://syngene.com) after ethidium bromide staining. The intensities of ∼180 bp fragments were quantified using image analysis software (Syngene). DNA laddering in the samples aged for 0, 25, 31 and 55 d time points has been previously reported by Kranner *et al*. [4], [14]. Here re-analysis of the same samples along with analysis of the early ageing time points (8, 10, 12, 13 and 15 d) was performed to show the entire ageing time course and to link the datasets together.

### HPLC analysis of low molecular weight thiol-disulphide redox state

Five replicates of 50 mg of ground seeds were analysed as described by Kranner and Grill [18] using a gradient, reversed phase HPLC (Jasco, http://www.jasco.co.uk) method with a C18 column (4.6 mm ID, 250 mm length; Kromatek, http://www.kromatek.co.uk). The half-cell reduction potentials (*E*_LMW disulphide/2LMW thiol_) were calculated using the Nernst equation [4], [19], and the redox environment determined according to Schafer and Buettner [20].

### Determination of glutathione reductase and glucose-6-phosphate dehydrogenase activities

Fifty mg ground material was extracted in 80% chilled acetone. For the measurement of glutathione reductase (GR) activity, glutathione disulphide (GSSG) and NADPH were added to the extract, and the oxidation of NADPH (extinction coefficient: 6.22 mM^−1^cm^−1^) was followed at 340 nm using a spectrophotometer (Jasco). The activity of glucose-6-phosphase dehydrogenase (G6PDH) was determined by following the reduction of NADP*^+^* at 340 nm after the addition of glucose-6-phosphate to the extract [21]. Data were expressed in nkat where one katal is defined as the amount of enzyme that catalyses the oxidation of 1 mol NADPH s^−1^ (for GR) or the reduction of 1 mol NADP^+^ s^−1^ (for G6PDH).

### Microarray analysis of gene expression

For each biological replicate (n = 3), one µg of total RNA was amplified and aminoallyl-labelled using a MessageAmp™ II aRNA Amplification kit (Ambion, http://invitrogen.com), and 7.5 µg of cRNA were labelled with either Cy3 or Cy5 Mono NHS Ester (Cy™Dye Post-labelling Reactive Dye Pack, GE Healthcare Life Sciences, http://www.gelifesciences.com). The samples were purified using Megaclear™ (Ambion) and Cy3 and Cy5 incorporation was measured using a NanoDrop spectrophotometer (Thermo Fisher Scientific, http://www.thermofisher.com). Labelled cRNA was fragmented using RNA Fragmentation Reagent (Ambion). The integrity and average size of total RNA, cRNA and fragmented cRNA were evaluated using a Bioanalyzer 2100 (Agilent Technologies, http://www.agilent.com). The average size of fragmented cRNAs was about 100 nucleotides. The probe was diluted to a final volume of 100 µl with hybridization solution.

Ps6kOLI1 microarrays (ArrayExpress accession number A-MEXP-142) contained 5,246 70-mer oligonucleotide probes representing 5,220 EST-clusters derived from 11,930 pea ESTs [22]. Each probe was represented by three replicate spots per microarray.

200 pmol of Cy5 and Cy3 aRNA fragmented probes were mixed with 20 µg of PolyA RNA (Sigma-Aldrich, http://www.sigmaaldrich.com) and 20 µg of yeast tRNA (Sigma-Aldrich) in a final volume of 90 µl of hybridization buffer (50% formamide, 6X SSC, 0.5% SDS, 5X Denhardt's). The probe was denatured at 95°C for 5 min and applied to the slide using a LifterSlip (Thermo Fisher Scientific, http://thermofisher.com). Slide hybridization, washing, drying and image capture were performed as recommended by the manufacturers.

Background correction and normalization of expression data were performed using LIMMA [23], [24] which is part of Bioconductor, an R language project [25]. The resulting log-ratios were print-tip loess normalized for each array [23]. To obtain similar distribution across arrays and to achieve consistency between arrays, log-ratio values were scaled using the median-absolute-value as scale estimator [23]. Linear model methods were used for determining differentially expressed genes. Each probe was tested for changes in expression across replicates by using an empirical Bayes moderated t-statistic [24]. *P*-values were corrected to control the false discovery rate (FDR) [26]. The expected FDR was found to be less than 5%, or 10% where specified. Only reproducible expression values (present in at least two gene replicates and FDR<0.05) were considered and log_2_ ratios were averaged. The hierarchical cluster was calculated and drawn using the TIGR MeV (Multiarray experiment viewer, version 4.4) software [27].

Microarray data have been deposited in the NCBI's Gene Expression Omnibus (GEO) [28] and are accessible through GEO Series accession number GSE24864 (http://www.ncbi.nlm.nih.gov/geo/query/acc.cgi?acc=GSE24864).

### cDNA synthesis and quantitative real-time PCR analysis

One µg of pea seed RNA was spiked with 100 ng of human RNA as an artificial internal control template and subsequently reverse transcribed using the Transcriptor First Strand cDNA Synthesis Kit (Roche, http://roche-applied-science.com). Two µl of cDNA were subjected to qRT-PCR using a LightCycler 2.0 Instrument (Roche), according to the LightCycler FastStart DNA Master^Plus^ SYBR Green I instructions (Roche). Amplification of the human porphobilinogen deaminase (*PBGD*) gene using *PBGD*-specific primers (Transcriptor First Strand cDNA Synthesis Kit, Roche) resulted in a 151 bp amplicon that was used as a reference gene. The sequences of pea-specific primer pairs used for qRT-PCR are given in Table S1. The following parameters were used for amplification: 1 cycle at 95°C for 10 min, 45 amplification cycles at 95°C for 10 s, 60°C for 10 s and 72°C for 10 s. The raw fluorescence data from each individual amplification reaction generated by the LightCycler software were used to calculate PCR efficiency (*E*) and cycle threshold (*Ct*) values using PCR Miner (http://www.miner.ewindup.info/) as described by Zhao and Fernald [29]. The efficiency-corrected expression ratio of the target genes relative to the *PBGD* reference gene was calculated using the following equation (modified after Pfaffl) [30]: *Ratio*  =  *E_target_^Ct control – Ct sample^* / *E_reference_^Ct control – Ct sample^*

Five biological replicates were used for all qRT-PCR analyses. Statistical analysis was performed using GraphPad Prism 4.

## Results

### Viability loss and nucleic acid degradation during pea seed ageing

**Figure 1:**
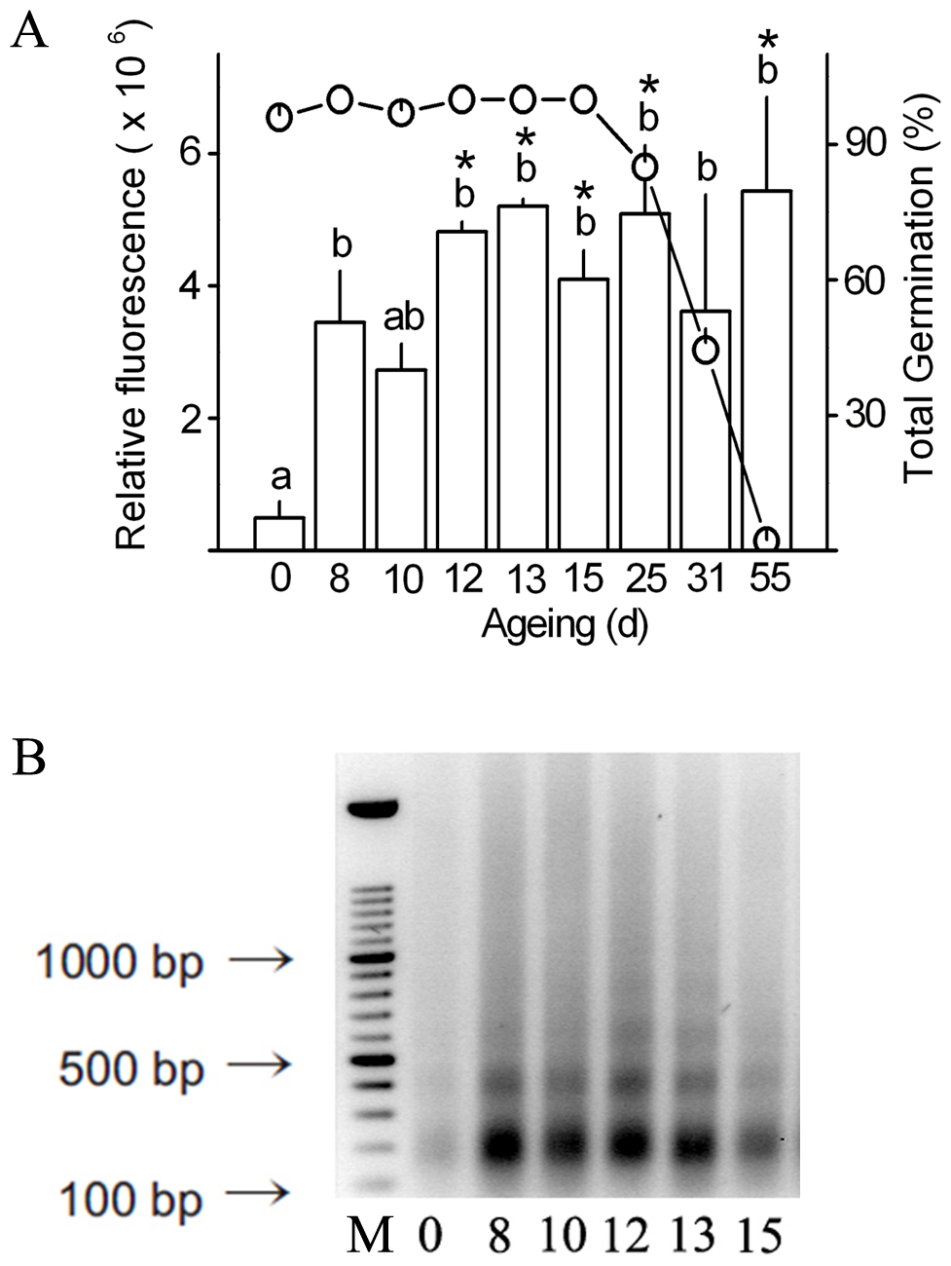
Fragmentation of DNA induced by ageing of pea seeds. (A) Semi-quantitative analysis of the 180 bp DNA fragments by image analysis. White bars (left y-axis) represent the mean ± SE relative fluorescence of the DNA fragments (n≥3), and circles (right y-axis) represent total germination (n≥3). Different letters indicate significant differences (*P*<0.05) in DNA integrity across all ageing treatments and asterisks indicate significant differences (*P*<0.01) between non-aged and aged seeds. (B) Gel electrophoresis of DNA from seeds. Lane ‘M’ shows a 100 bp molecular mass marker (Roche) and the other lanes represent one of at least three independent biological replicates; the ageing period (in days) is shown at the bottom of each lane.

Pea seeds were subjected to artificial ageing (“ageing” at 12% MC and 50°C) for up to 55 d, and viability was assessed by germination testing. High viability was maintained [96% to 100% total germination (TG)] during the first 15 days of ageing, and subsequently declined to 2% TG after 55 d (Figure 1A). Nucleic acid degradation is a marker of seed ageing, and DNA and RNA integrity were assessed in ageing pea seeds. There was little DNA fragmentation in non-aged control seeds, but ageing resulted in the appearance of “DNA ladders”, composed of bands of ∼180 base pairs and multiples thereof (Figure 1B). Electropherograms of RNA showed the gradual disappearance of distinct 18S and 25S rRNA peaks, increasing baseline noise and a decrease in the RNA integrity number (RIN; Figure S1) during seed ageing; indicative of RNA degradation. The effects of ageing were clearly evident from the earliest time point (8 d), however, no loss of viability was observed until after 25 d of ageing, by which point RNA was almost completely degraded.

### Determination of low molecular weight thiol and disulphide concentrations and half-cell reduction potentials and the activities of glutathione reductase and glucose-6-phosphate dehydrogenase

**Figure 2:**
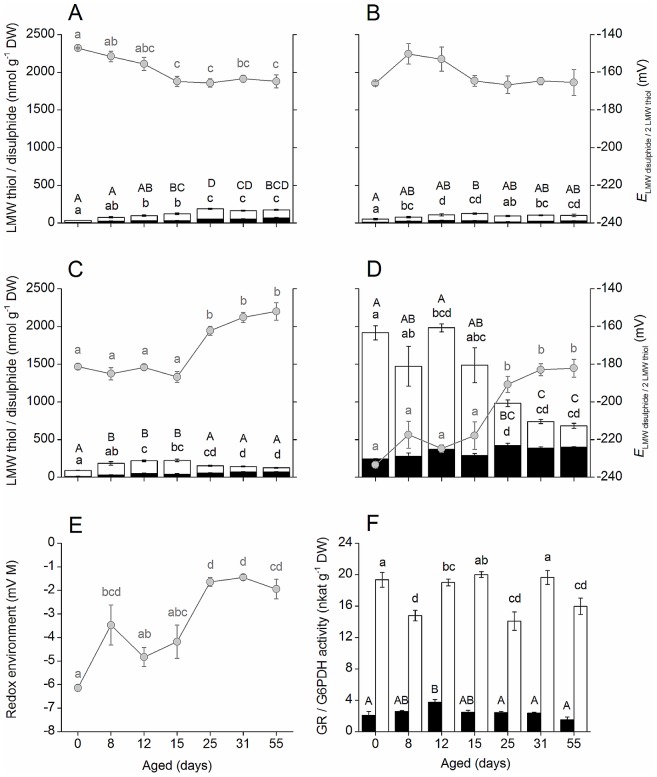
The effect of ageing on the redox state of low molecular weight thiol-disulphide couples and the activities of glutathione reductase (GR) and glucose-6-phosphate dehydrogenase (G6PDH). (A – D) Concentration of low molecular weight thiols (white bars; A: cysteine; B: cysteinyl-glycine; C: γ-glutamyl-cysteine; D: glutathione) and corresponding disulphides (black bars) at each ageing interval (left-hand y-axis). Bars represent means ± SE. (n = 4 or 5). Grey circles represent the half-cell reduction potential ± SE (n = 4 or 5) of each redox couple (right-hand y-axis; A: *E*_cystine /2 cysteine_; B: *E*_Cys-bis-Gly/2 Cys-Gly_; C: *E*_γ-Glu-bis-Cys/2 γ-Glu-Cys_; D: *E*_GSSG/2 GSH_). Different letters indicate significant differences across all ageing time points (one-way ANOVA; Tukey's test, *P*<0.05; black upper case for thiols, black lower case for disulphides and grey lower case for half-cell reduction potentials). (E) Overall redox environment defined by the cysteine / cystine, cysteinyl-glycine / bis-cystinyl-glycine, γ-glutamyl-cysteine / γ-glutamyl-bis-cystine and GSH / GSSG redox couples represented by grey circles ± SE (n = 4 or 5). Different letters indicate significant differences (one-way ANOVA; Tukey's test, *P*<0.05) across all ageing time points. Ageing resulted in a shift of *E*_GSSG/2GSH_, towards more positive values, indicating more oxidizing conditions; values more positive than −180 mV have previously been related to viability loss. (F) Activities of GR (white bars) and G6PDH (black bars). Bars represent means ± SE (n = 4 or 5). Different letters indicate significant differences (One-way ANOVA; Tukey's Test, *P*<0.05, lower case for GR and upper case for G6PDH) across all ageing time points.

Accumulation of ROS and oxidative damage are key components of the ageing process [3], [4]. Therefore, analysis of the oxidative status of *P. sativum* seeds during ageing was undertaken by measuring the changes in the concentration and redox state of low molecular weight thiols (cysteine, cysteinylglycine, γ-glutamylcysteine and glutathione; Figure 2). There were significant increases in the concentrations of cysteine and cystine from 15 d and 12 d, respectively, which resulted in a significant shift in the half cell reduction potential of the cysteine/cystine redox couple (*E*_cystine/2Cys_) to more negative (more reducing) values after 15 d of ageing (Figure 2A). There were only slight changes in the concentrations of cysteinylglycine during ageing, whilst cystinyl-bis-glycine was increased from the onset of ageing. However, there was no signficant change in *E*_Cys-bis-Gly/2Cys-Gly_ (Figure 2B). γ-Glutamylcysteine increased from the onset of ageing, before levels declined after 25 d. In contrast, the concentration of bis-γ-glutamylcystine increased throughout the ageing period. *E*_bis-γ-Glu-Cys/2γ-Glu-Cys_ was not significantly affected until after 25 d ageing, when it shifted towards more positive values (Figure 2C). The major low molecular weight thiol was glutathione and in non-aged seeds the concentration of total glutathione [reduced glutathione (GSH) + glutathione disulphide (GSSG)] was 1.9 µmol g^−1^ dry weight, and comprised 11% GSSG. No significant change in GSH concentration occurred until 25 d when there was a 50% drop in GSH concentration (Figure 2D). In contrast, the concentration of GSSG increased by 74% after 25 d ageing. After 55 d the total glutathione concentration was approximately half that of non-aged seeds, and 37% comprised GSSG. *E*_GSSG/2GSH_ did not change significantly during the first 15 d of ageing, but increased towards more positive (more oxidizing) values as seeds aged further (25 d, 31 d and 55 d; Figure 2D). The overall thiol-defined redox environment was calculated by summing the products of the reduced thiol concentration and the corresponding half-cell reduction potential for each redox couple. In non-aged seeds the redox environment was -6.15 mV M and this shifted to −3.48 mV M after 8 d of ageing, and shifted further to −1.64 mV M after 25 d of ageing when seeds started to lose viability. GSH/GSSG was the most abundant redox couple and therefore had the greatest influence on the redox environment, accounting for almost 94% in non-aged seeds. However, as GSH declined during ageing, the relative contribution of the other low molecular weight thiol-disulphide couples to the overall redox environment increased from 6% in non-aged seeds to 43% in seeds aged for 55 d (Figure 2E).

The activities of glucose-6-phosphate dehydrogenase (G6PDH) and glutathione reductase (GR) were measured to gain further insights into the relationship between oxidative stress and ageing. G6PDH activity was unaltered during ageing, whilst the activity of GR was lower in aged seeds (*P*<0.05) compared with non-aged seeds at most ageing time points (Figure 2F).

### Global gene expression profiling during the early stages of seed ageing

**Figure 3:**
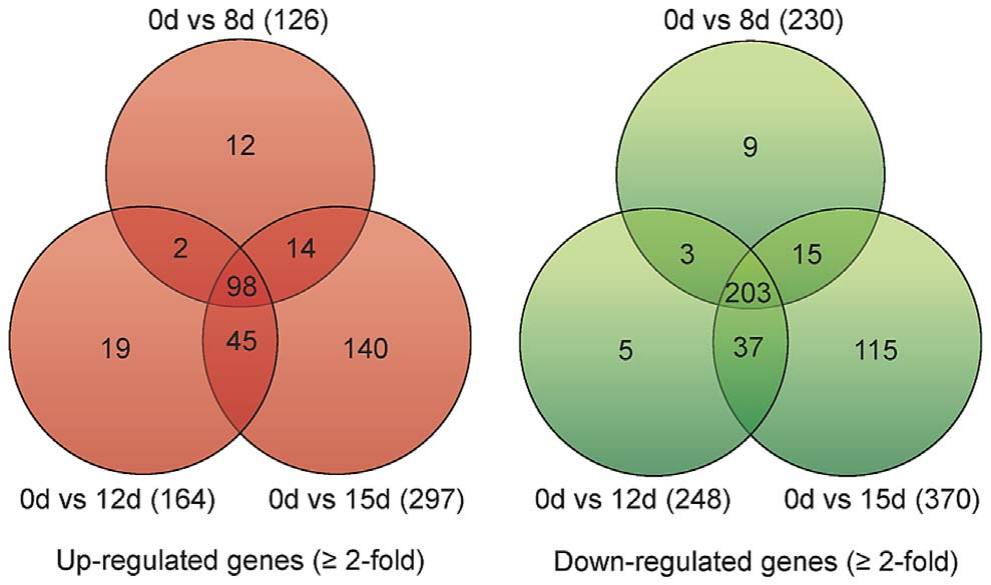
Venn Diagrams showing the numbers of genes up- and down- regulated during ageing of pea seeds. Values in brackets represent the total number of genes that are up- or down-regulated (≥2-fold change in expression compared to non-aged controls; n = 3) at a specific ageing time-point (8 d, 12 d, and 15 d).

**Figure 4:**
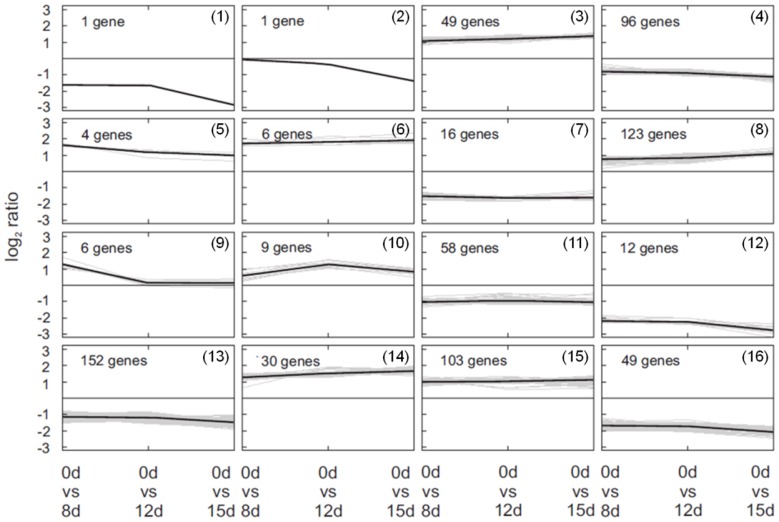
Expression profiles of ageing-responsive gene clusters. Genes that were differentially expressed (≥2-fold change in expression compared to non-aged controls) at one or more time points (8 d, 12 d and 15 d) during ageing of *Pisum sativum* seeds were clustered using TIGR Multi-experiment Viewer, version 4.4 [27]. Clustering was performed on the basis of similarities between gene expression profiles throughout the ageing time course. The mean expression values for each gene at each of the three ageing time points (8 d, 12 d and 15 d) were normalized to the mean expression in the non-aged controls (0 d). log2 (ratio) was used to represent expression levels. Clusters 1 and 2 contain only one gene due to their unique expression profiles during the ageing process. Three biological replicates were used for each transcriptomic comparison. Values in brackets correspond to the cluster numbers used in the text and Table S3.

**Figure 5:**
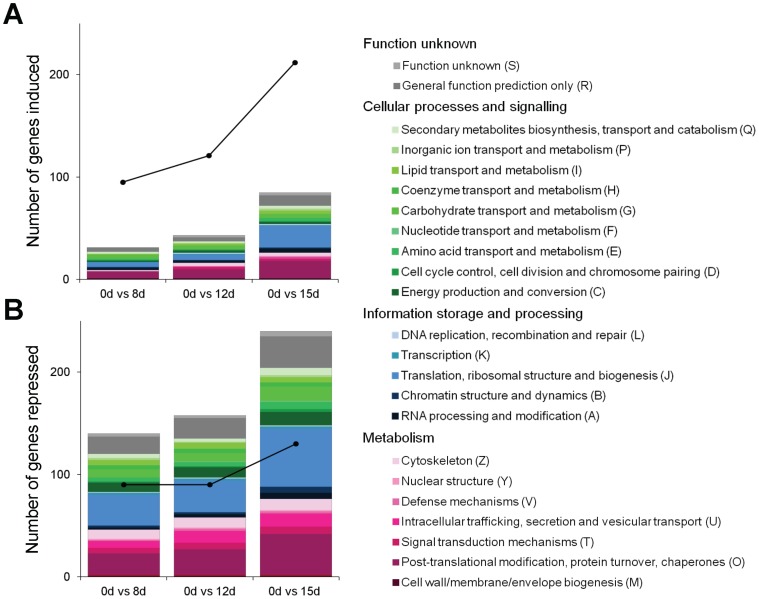
Numbers of genes up- and down-regulated during ageing of pea seeds according to KOG functional category. (A) Up-regulated genes (log_2_ ratio ≥1.0); (B) down-regulated genes (log_2_ ratio ≤-1.0). Stacked bars represent the numbers of genes induced or repressed after 8 days, 12 and 15 days of ageing (n = 3). Colours represent different KOG classes (grey, function unknown; green, cellular process and signaling; blue, information storage and processing; purple, metabolism) and the different shades correspond to the KOG functional categories within each class. The black circles and lines represent the numbers of induced or repressed genes that were not classified according to the KOG database.

Microarray profiling of gene expression of non-aged control seeds and seeds aged for 8, 12 and 15 days was performed (Figures 3, 4, 5 and S2, and Tables S2–S4). A total of 717 differentially expressed genes were identified during the ageing process [≥2-fold change in expression (log_2_ ratio ≥1.0 or ≤−1.0) with a false discovery rate (FDR) <0.05], 330 of which were up-regulated and 387 were down-regulated (Figure 3, S2 and Table S2). There was strong correlation between the gene expression profiles of the three ageing intervals (Figure S3). The most drastic changes in expression occurred after 15 d of ageing, with 140 and 115 genes specifically up-regulated and down-regulated, respectively (Figure 3). All 717 differentially regulated genes were clustered into 16 ageing-responsive expression patterns (Figures S2 and 4 and Table S3). The majority (68%) of up-regulated genes belonged to clusters 8 and 15, whereas down-regulated genes belonged mainly to clusters 4 and 13. Clustered genes may be co-regulated e.g. induced or repressed by the same transcription factors. Approximately half of the differentially expressed genes were assigned a functional annotation according to the euKaryotic Orthologous Groups (KOG) database, whilst 70% and 40% of up-regulated and down-regulated genes, respectively, were of unknown function. The annotated genes were divided into major functional categories according to the KOG classification (Figure 5, Table S2).

Up-regulated genes belonged to 21 functional KOG categories, whilst down-regulated genes were divided into 23 functional categories. At each ageing time point the majority of up- and down-regulated genes were associated with ‘protein post-translational modification, turn-over and chaperones’, and ‘translation, ribosomal structure and biogenesis’ (Figure 5). Many of the genes associated with ‘post-translational modification, protein turnover and chaperones’ encode proteins involved in the ubiquitin-proteasome pathway. Also within this category were genes encoding proteins involved in redox modification of proteins, such as thioredoxin-h, peroxiredoxin, glutaredoxin and glutathione peroxidase and a number of heat shock proteins. Other genes within the ‘cellular processes and signalling’ class included several calcium signalling genes: two calmodulin genes and a calcium-dependent protein kinase (CDPK) were repressed, whilst another CDPK was up-regulated. CDPKs are involved in stress response [31] and a CDPK protein was also up-regulated during artificial ageing of maize seeds [32].

The vast majority of genes associated with ‘translation, ribosomal structure and biogenesis’ encoded 40S and 60S ribosomal proteins, and around 75% were down-regulated during ageing (Tables S2 and S4). Rajjou *et al*. [33] also reported a decline in proteins involved in translation during seed ageing and suggested that this caused protein synthesis during germination to be delayed to allow repair of nucleic acid damage prior to translation. Other genes within the ‘information storage and processing’ class included genes involved in RNA processing and modification, of which two genes encoding aconitases, and an RNA helicase were induced by ageing. Nine chromatin structure genes, mainly encoding histone proteins, were also differentially expressed during ageing. Few genes involved in transcription or DNA replication and repair were ageing-responsive.

Twenty ageing-responsive genes were associated with ‘carbohydrate transport and metabolism’, of which nine encoded glycolytic enzymes, with five up-regulated during ageing (Table S2). Sucrose synthase was strongly up-regulated (log_2_ ratio ≥1.5) at early stages of ageing and hexokinase was also up-regulated. Two genes associated with ‘energy production and conversion’ were up-regulated during ageing: 12-oxophytodienoate reductase (*OPR1*), which is involved in jasmonic acid biosynthesis and also up-regulated during *Arabidopsis* leaf senescence [34], and dihydrolipoyl dehydrogenase. Other metabolic genes affected by ageing included genes associated with the transport and metabolism of lipids, amino acids, inorganic ions, coenzymes and nucleotides (Table S2). Two genes involved in control of the cell cycle and cell division were down-regulated during ageing and one gene encoding cullin-1 was up-regulated. Cullin-1 forms part of the SCF E3 ubiquitin ligase complex that mediates the ubiquitination of cell cycle proteins [35]. Patellin-5, which was the most strongly induced gene, is also involved in the cell cycle through membrane trafficking. Other strongly induced genes included glutathione-dependent formaldehyde dehydrogenase (FALDH), cysteine proteinase, two universal stress protein family genes and a legumin J precursor (Table S2). Legumins are seed storage proteins, and of seven seed-specific genes up-regulated during ageing, four encoded legumins (Table S4).

### Ageing-responsive expression of genes related to PCD and oxidative stress

Since DNA fragmentation, which is a hallmark of PCD, was observed from the earliest ageing time point (8 d; Figure 1B), and oxidative stress has been linked with seed ageing [4], special attention was given to genes involved in PCD and oxidative stress response. A large proportion of differentially expressed genes were not KOG annotated, but many were assigned functional annotations according to the UniProt Knowledgebase, which enabled their inclusion in the functional categories shown in Table S4.

Twenty-three genes with predicted PCD-related functions showed differential expression during ageing, of these 15 were up-regulated, including Bax inhibitor 1 (*BI-1*), NIMA-related kinase, MA3-domain containing protein, two dehydration-responsive protein precursors (*RD22*), DNA-damage inducible protein (DDI1-like protein), knotted-like protein 4 (*KNAT4*), two cysteine proteinases (papain and RD21), aconitases, a putative sentrin-specific protease and dihydrolipoyl dehydrogenase (Table S4). All have been previously reported to be up-regulated during PCD triggered by various stresses e.g. ionizing radiation [36], apoptosis [37], and desiccation, salt and ABA treatments [38]. Half of the up-regulated PCD-related genes belonged to cluster 8, and were up-regulated only at the later ageing time point of 15 d. All eight down-regulated genes were repressed by 8 d of ageing, and further repressed after 15 d of ageing, with half belonging to cluster 13 (Figure 4 and Table S3).

Ageing modulated the expression of 15 oxidative stress-related genes (Table S4). Four genes were up-regulated, and encoded two FALDHs, a putative peroxidase and a thioredoxin *h*. FALDH exhibits S-nitrosoglutathione reductase activity, and may play a role in nitric oxide homeostasis [39]. Down-regulated genes included peroxidase 42, monodehydroascorbate reductase, glutathione peroxidase, glutathione-S-transferase and a glutaredoxin-related protein. Other genes that may be oxidative stress-responsive included two ferritin-3 genes, which were induced during seed ageing.

Twenty-eight ubiquitin-proteasome genes were affected by ageing, of which 11 genes were up-regulated. Most genes were induced within 8 d of ageing (cluster 3 and 15) and their expression increased with ageing period, indicating that these genes may play a role in activating ageing-responsive pathways. Four genes were induced only after 15 d of ageing (cluster 8), and may be involved in the ubiquitination of proteins for degradation as part of the execution phase of PCD (Table S4).

### Analysis of gene expression throughout the complete ageing time course by qRT-PCR using an artificial reference gene

The assessment of gene expression during seed ageing is fraught with difficulties due to the degradation of nucleic acids, leading to an apparent down-regulation of overall gene expression levels, including housekeeping genes [40]. In addition, many commonly used housekeeping genes are not stably expressed in seeds [41]. Our microarray analysis showed that 21 commonly used housekeeping genes were differentially expressed during seed ageing (Table S4). To address this problem pea RNA was spiked with total human RNA and the human-specific porphobilinogen deaminase (*PBGD*) gene was used as an artificial reference gene to normalize the expression of pea-specific target genes using qRT-PCR (see Figure S4 for an overview) [42], [43], [44]. *PBGD* expression was detected only in pea samples spiked with human RNA and there were no detectable signals for any of the target *P. sativum* genes when total human RNA was used exclusively as template, nor did the addition of total human RNA to the pea samples change the non-normalised expression of any of the target *P. sativum* genes compared to non-spiked samples.

**Figure 6:**
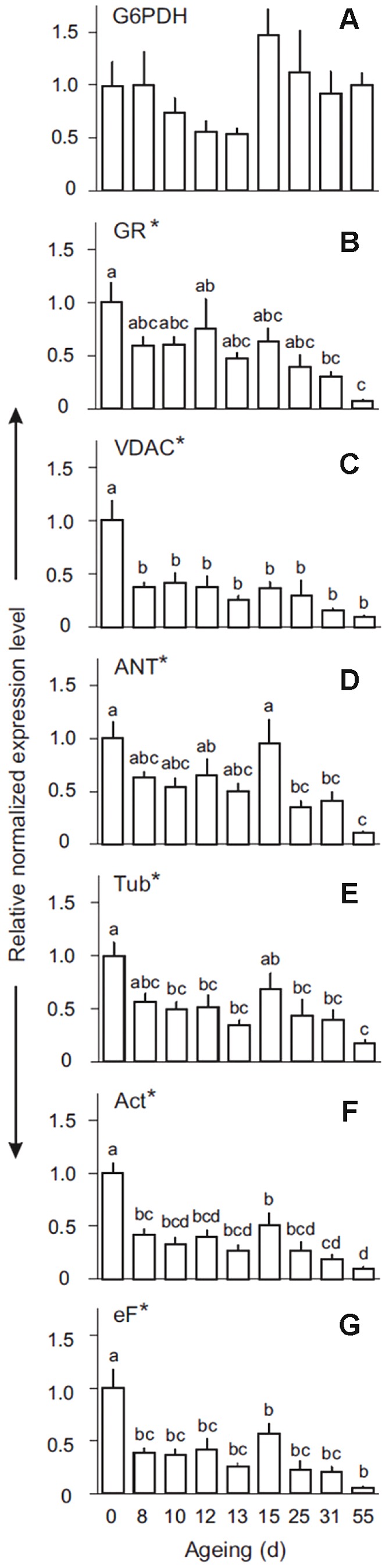
Normalised expression of selected genes during seed ageing. Expression of glucose-6-phosphate dehydrogenase (*G6PDH*), glutathione reductase (*GR*), voltage-dependent anion channels (*VDAC*), adenine nucleotide translocator (*ANT*), β-Tubulin 3 (*Tub*), Actin 1 (*Act*) and elongation Factor-1α (*eF*) was analysed using qRT-PCR. Data were normalised using the spiked human *PBGD* gene and changes in expression following ageing are shown relative to the non-aged control. Bars represent means + SE (n = 5). Different letters indicate significant differences (one-way ANOVA; Tukey's test, *P*<0.05) across all ageing time points. Asterisks denote genes with a regression slope deviating significantly from zero (linear regression analysis, *P*<0.05, R^2^: *G6PDH*  = 0.02610; *GR*  = 0.8296; *VDAC*  = 0.5265; *ANT*  = 0.6664; *Tub*  = 0.5899; *Act*  = 0.5293; *eF*  = 0.5565).

The expression of two glutathione-related genes encoding glutathione reductase (GR) and glucose-6-phosphate dehydrogenase (G6PDH), two potentially PCD-related genes encoding voltage-dependent anion channel (VDAC) and adenine nucleotide translocator (ANT) and three commonly used house-keeping genes [actin 1 (*Act*), tubulin-beta-3 (*Tub*), elongation factor-1 alpha (*eF*)] in seed samples incrementally aged for up to 55 days were investigated using qRT-PCR. Linear regression analysis was used to determine stably expressed genes during ageing. The artificial *PBGD* reference clearly displayed the smallest overall variation (Figure S5). *G6PDH* expression was unaltered during ageing, whilst *GR, VDAC, ANT, Tub, Act, eF* were all down-regulated (Figure 6; Figure S5). The down-regulation of house-keeping genes confirmed that the artificial *PBGD* reference gene was a more appropriate normalisation factor. The expression patterns observed using qRT-PCR generally confirmed the microarray data, with the exception of *GR*, which was unaltered in the microarray analyses, and *ANT*, which was repressed from the earliest ageing time point in the microarray study, but not significantly repressed until 25 d ageing in the qRT-PCR analysis. For all genes analysed, with the exception of *G6PDH*, expression declined further at the later stages of ageing (after 25 d), and was lowest in seeds aged for 55 d, which is likely to reflect the widespread RNA degradation at these time points.

## Discussion

### An increase in glutathione half-cell reduction potential coincides with the onset of viability loss

*E*_GSSG/2GSH_ is proposed to be a marker of cell viability [4], [20], and the dramatic increase in *E*_GSSG/2GSH_ after 25 d ageing coincided with the onset of viability loss (Figure 1A and 2D), as previously reported by Kranner *et al*. [4], and is indicative of progressive oxidative stress during ageing. This work complements the earlier study [4], by analysing changes in half-cell reduction potentials of low molecular weight thiols throughout the ageing time course, including the early stages of ageing prior to the onset of viability loss, which were not previously reported. *E*_bis-γ-Glu-Cys/2γ-Glu-Cys_ followed a similar pattern to *E*_GSSG/2GSH_, whilst neither *E*_cystine/2Cys_ nor *E*_Cys-bis-Gly/2Cys-Gly_ became more positive during ageing of pea seeds. Probit analysis was used to estimate that 50% of seeds lost viability when *E*_GSSG/2GSH_ and *E*_bis-γ-Glu-Cys/2γ-Glu-Cys_ reached −187 to −182 mV and −159 to −156 mV, respectively, which are similar to the values predicted for artificially aged *Lathyrus pratensis* seeds [19]. The low molecular weight thiol-defined cellular redox environment became more oxidising as ageing progressed, and 50% of seeds lost viability when the redox environment reached values of −1.45 to −0.77 mV M. A similar pattern was observed in artificially aged *Lathyrus pratensis* seeds, although the zone of viability loss was estimated to be narrower and slightly more positive (−1.03 to −0.69 mV M), which likely reflects inter-species variation in the dynamics of thiol-disulphide turn-over.

The decline in GSH as ageing progressed could be due to degradation leading to the formation of cysteinylglycine and cysteine, both of which increased slightly during seed ageing, but not enough to account for the decline in GSH levels. An alternative fate for GSH is the *S*-glutathionylation of protein Cys residues, which occurs under conditions of oxidative stress and may serve to protect protein thiols from irreversible oxidation. *S*-Glutathionylation can directly influence the activity of proteins, and targets include glycolytic enzymes, signalling proteins and proteins involved in redox homeostasis [45]. *S*-Glutathionylation has also been reported to be an early event in the induction of cell death [46].

GR plays an important role in maintaining a high ratio of GSH/GSSG in cells [47], [48], [49], and activity decreased during ageing. A similar response was observed following treatment with high concentrations of H_2_O_2_ [50], which suggests that ROS accumulation during ageing could cause reduction in GR activity, leading to a decreased GSH/GSSG ratio. PCD is associated with GSH/GSSG imbalance, and a rise in GSSG has been suggested to precede the loss of mitochondrial integrity, cytochrome c release and caspase-3 activation [4], [51]. Glutathione redox state has also been reported to influence the activity of the ubiquitin/26S protein degradation pathway [52], which was shown in this study to play an important role in ageing of pea seeds (Table S4), and previously noted to be involved in senescence [53].

### DNA laddering indicates the involvement of programmed cell death in seed ageing

RNA degradation [54] and DNA fragmentation are associated with PCD, and although PCD may occur in the absence of both [55], [56]; there are few reports of DNA laddering occurring under any circumstances apart from PCD [57]. Therefore, this indicates that PCD was initiated from the very beginning of ageing. The up-regulation of 46% of differentially expressed genes indicates the occurrence of *de novo* transcription, so apparent changes in expression were not solely due to degradation of stored mRNAs. The moisture content of the pea seeds was 12%, and at 50°C the cytoplasm, although highly viscous, is not in a glassy state, which would facilitate the occurrence of cellular processes including transcription. The decline in transcript abundance of 387 genes may result from transcriptional regulation, but degradation of stored mRNA may also contribute to the decline. The mechanisms of selective mRNA degradation have yet to be determined, but may depend on the inherent sensitivity of specific mRNAs to oxidation [12].

### A decline in antioxidant capacity may facilitate the progression of programmed cell death signalling

The down-regulation of several antioxidant genes is possibly due to mRNA degradation, and a similar decline was reported for catalase transcript levels during ageing of sunflower seeds [58]. Expression of genes encoding antioxidants or proteins involved in repair of oxidative damage may be repressed as part of the PCD process, to allow cell death to proceed rapidly. He and Kermode [59] reported that H_2_O_2_ production was required for activation of caspase-like proteases, and that antioxidants slowed the progression of cell death. Furthermore, inhibition of the degradation of catalase by the ubiquitin-proteasome pathway also slowed cell death, suggesting that selective degradation of antioxidant enzymes provides a means of regulating ROS production and PCD. Nitric oxide and protein nitrosylation may play a role in PCD by modulating antioxidant activity [60]. Some oxidative stress-related genes were induced during seed ageing. For example ferritins, which bind iron and prevent ROS formation via the Fenton reaction [61], and thioredoxin *h*, which may be induced by oxidative stress [62], and is involved in the regulation of protein thiolation. The thioredoxin and glutaredoxin systems are reported to be involved in apoptosis-like PCD [63], [64]. Reduced thioredoxin binds to apoptosis signal-regulating kinase 1 (ASK1) to inhibit downstream signalling pathways. Glutaredoxins and peroxiredoxins may also act in a similar way through interaction with the thioredoxin-binding domain of ASK1 [65], [66]. Other negative regulators of ASK1 include 14-3-3 proteins [67]. Two genes encoding 14-3-3-like proteins were altered in expression during seed ageing, one was up-regulated and the other down-regulated. ROS and oxidative stress cause dissociation of inhibitor proteins to activate ASK1 and initiate downstream MAP kinase signalling pathways leading to stress responses and PCD [68].

### Seed ageing causes endoplasmic reticulum stress

PCD in animals and plants shares some common phenomena, such as the release of apoptogenic proteins (e.g. cytochrome c) from the mitochondria [69], induction of caspase-like proteases, and DNA cleavage by PCD-active nucleases to yield multimers of approximately 180bp [70], [71]. In animal cells regulation of the mitochondrial PCD pathway involves proteins of the B-cell lymphoma 2 (Bcl-2) family e.g. Bax which induces release of cytochrome c from the mitochondria and Bcl-2, which inhibits Bax. Neither protein has been identified in plants, although the heterologous expression of mammalian Bax in *A. thaliana* and tobacco causes PCD, which is reversed by the over expression of Bax inhibitor-1 (*BI-1*) [72], [73], [74]. BI-1 is localised in the endoplasmic reticulum (ER), and controls calcium flux into and out of the ER [60], possibly through interaction with calmodulin [75]. Expression of *BI-1* is up-regulated by pathogen inoculation [76], [77] and senescence [72], [78], [79]. In this study *BI-1* was up-regulated after 8 days of seed ageing and is likely to play a role in suppressing PCD and maintaining seed viability (Table S4). BI-1 is also up-regulated in response to water deficit, and suppresses ER stress-induced PCD [80].

Conditions of oxidative stress disturb the protein folding environment of the ER lumen leading to accumulation of unfolded and misfolded proteins. This triggers the unfolded protein response (UPR), which mitigates ER stress by up-regulating the expression of chaperones and co-chaperones of the protein folding machinery [81]. Binding protein (BiP) is the most abundant chaperone of the ER and its up-regulation is considered to be a marker of ER stress [82]. In this study *BiP2* expression was induced after 12d, which indicates the occurrence of ER stress during seed ageing. Derlins may be involved in the targeting of misfolded proteins to the cytosol for ubiquitination [83]. Two derlin 2.2 genes were differentially expressed during seed ageing, indicating that ER-associated degradation is affected by ageing. The ubiquitin/26S proteasome system degrades misfolded proteins, 28 ubiquitin-proteasomal genes were altered in expression during ageing, indicating that this is a key ageing-responsive pathway. The ubiquitin-proteasomal system may play a vital role in the degradation of damaged proteins, such as those irreversibly oxidised during seed ageing [33]. In addition, ubiquitination may promote PCD through the targeted degradation of negative regulators of PCD by specific E3 ligases [84].

### Mitochondrial release of cytochrome c via the permeability transition pore is repressed at the transcription level

Multiple stimuli cause up-regulation of cytochrome c and cytochrome c oxidase expression as early events of PCD [85]. However, during seed ageing, the expression of both genes was down-regulated, and showed a similar trend to that observed in senescing leaves [78]. Increases in cytosolic calcium induce release of cytochrome c from mitochondria through activation of the permeability transition pore (PTP), which consists of VDAC and ANT [86]. Up-regulation of *VDAC* has been reported in plant PCD in response to drought, cold and salicylic acid [87], *Pseudomonas syringae* [88] and also during the plant hypersensitive response [89]. In ageing seeds *VDAC* was down-regulated (Table S4) and the same occurred during leaf senescence [78], which indicates that *VDAC* may play a specific role in ageing-induced PCD that differs from PCD triggered by other stresses. *ANT* was also down-regulated in response to seed ageing (Table S4). Down regulation of *ANT* is an early event in the execution phase of PCD induced by heat and senescence [78] and the defence response in *Arabidopsis* [90]. A decline in ANT protein synthesis may result in a decrease in ATP/ADP exchange, which is one of the earliest events of PCD [91]. Other genes that may play a role in the release of pro-apoptotic mitochondrial proteins into the cytosol are aconitases and dihydrolipoyl dehydrogenases, which appear to be particularly susceptible to oxidative stress and were up-regulated in response to seed ageing, indicating a possible involvement in redox signaling to trigger PCD [92], [93], [94].

There have been several reports of a link between carbohydrate metabolism and PCD. For example, glyceraldehyde-3-phosphate dehydrogenase (GAPDH) has been linked to PCD in animal cells [95], [96]. GAPDH is inhibited by oxidation of a Cys residue in the active site, which can activate a MAP kinase signalling cascade via a multistep phosphorelay [97]. In addition, disulfide bond formation between GAPDH subunits leads to aggregation, which correlates with the rate of oxidative stress-induced cell death [98]. In this study several glycolytic genes including *GAPDH* were up-regulated. Likewise, Rajjou *et al*. [33] observed increased levels of GAPDH in a proteomic study of seed ageing, and phosphofructokinase expression was induced during silique wall senescence in *Arabidopsis* [79]. Both hexokinase and sucrose synthase were up-regulated during seed ageing, and induction of sucrose synthase activity was also reported during accelerated ageing of sunflower seeds [99]. Hexokinase is associated with the mitochondrial membrane, and may act as a glucose sensor and regulate mitochondrial ROS levels. In animal cells hexokinases may be involved in the control of PCD [100]. Hexokinase binds to the VDAC protein to induce closure of the PTP and prevent cytochrome c release, and a similar mechanism may operate in plants [96]. Sucrose synthase is involved in inter-compartmental signalling and also interacts with VDAC possibly operating via an analogous mechanism to hexokinase [101].

### Seed ageing causes modulation of cysteine proteases with caspase-like activity associated with programmed cell death

The critical point in the PCD pathway of animal cells is the activation of caspases [102], [103]. Metacaspases have been identified in plants, which share sequence and structural similarity with animal caspases, but do not have caspase-like activity [104]. However, increasing evidence indicates that cysteine proteases with caspase-like activity are induced during plant PCD [105], [106]. Here, in ageing seeds, two cysteine proteases, *RD21* and *papain*, were up-regulated (Table S4), which is consistent with the proposed caspase-like role of RD21 in plant PCD [107], [108]. In contrast, legumain, another cysteine protease with caspase-like activity [109] was repressed. Treatment with caspase inhibitors prior to ageing has been shown to reduce seed viability loss, which provides evidence for the involvement of caspases in seed ageing and death [4], [110]. Caspase-like activity has also been linked to the proteasome. For example, the proteasome subunit PBA1 has caspase-like activity [60] and the 20S proteasome is responsible for caspase-3 activity and involved in PCD during xylem development [111]. In plant cells, the proteasome may activate the caspase cascade to induce cell death, whilst in animal cells, the proteasome-mediated steps of apoptosis are located upstream of mitochondrial changes and caspase activation, possibly involving Bcl-2 among other proteins [112]. Caspases may be subject to redox control and caspase-3 is inhibited by *S*-glutathionylation [45], [113].

### Similarities between the seed ageing transcriptome and senescence suggest that there is overlap between the pathways leading to induction of PCD

Whilst ageing seeds showed activation of some PCD-related genes, others such as *VDAC*, cytochrome c and cytochrome c oxidase were repressed. Similar expression patterns have been observed in senescence-induced PCD [78], indicating that there is considerable overlap between ageing and senescence-induced PCD routes. However, in contrast to senescence, there was little up-regulation of antioxidant genes in ageing seeds, and no senescence-activated genes (SAGs) or senescence-related genes (SRGs) were differentially expressed. Other genes that showed a similar response during seed ageing to that reported during senescence included oxidative stress-related genes (glutathione-S-transferase, mitochondrial Mn superoxide dismutase, monodehydroascorbate reductase), transcription factors (NAC, MYB family), ubiquitin-proteasome pathway genes, heat shock genes (*HSP70*), HR–related genes, histone genes (*H2A*, *H2B* and *H3*), beta-tubulin genes, two calmodulin genes (*CaM7* and *CaM8*), 12-oxophytodienoate reductase (*OPR1*) [34], an FtsH protease and several kinases including a calcium-dependent kinase, serine threonine protein kinases and phosphofructokinase [78], [109], [114], [115].

**Figure 7:**
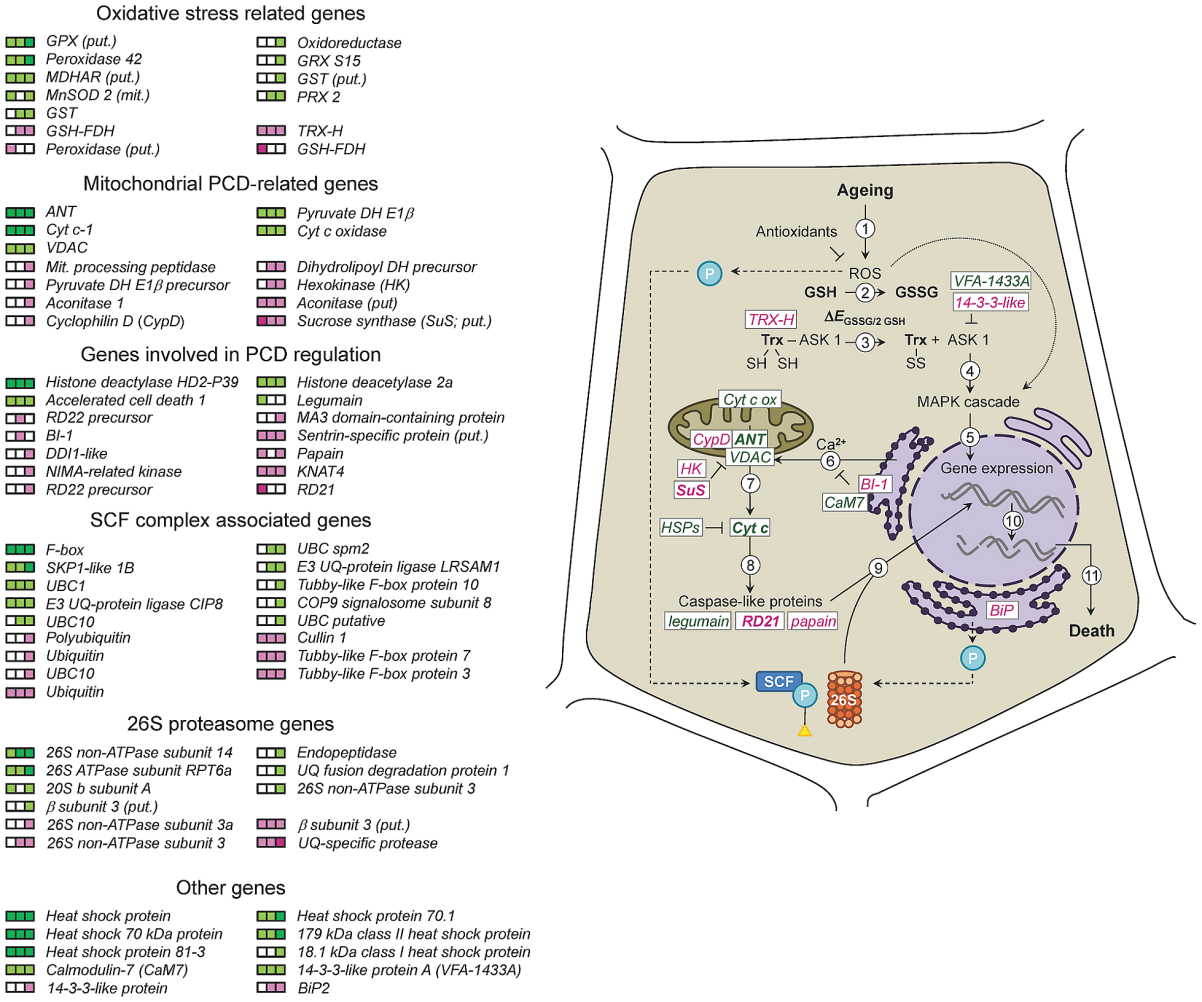
A model of events leading to programmed cell death during seed ageing. Differentially expressed PCD-related genes were mapped onto the model proposed by Kranner *et al*. [4] (solid arrows). ROS formed during ageing (1) are scavenged by GSH leading to an increase in *E*_GSSG/2GSH_ (2), which causes the apoptosis signal-regulating kinase (ASK 1) to be split from thioredoxin (Trx; *TRX-H*; 3). ASK1 activates a MAPK cascade (4) leading to changes in gene expression (5). ROS may also participate directly in signalling pathways to activate MAP kinase cascades (dotted arrow). Ca^2+^ is released from the ER (6), which induces release of cyt c from the mitochondrion (7). Cyt c activates caspases (8) and a downstream proteolytic cascade (9) which leads to the breakdown of structural and nuclear proteins and activation of nucleases which cleave DNA (10) as part of the execution phase of PCD leading to cell death (11). ROS cause protein oxidation, and the damaged and mis-folded (due to ER stress) proteins (P) are degraded by the ubiquitin-proteasome pathway (dashed arrows). The ubiquitin-proteasome has been linked with activation of the caspase proteolytic cascade and some proteasome subunits have caspase-like activity, which may offer an alternative route to PCD. The coloured blocks on the left-hand side indicate the ratio of expression of genes involved in the model at three different ageing time points (from left to right: 8, 12 and 15 days) to expression in non-aged seeds. Expression values corresponding to a log_2_ ratio ≥1.0 or ≤−1.0 are shaded in light magenta (up-regulation) or light green (down-regulation), and those which were changed by a log_2_ ratio of ≥1.5 or ≤−1.5 are shaded in dark magenta (up-regulation) or dark green (down-regulation). White blocks indicate no change in expression between aged and non-aged seed.

Senescence involves the active turnover and recapture of cellular material, and precedes widespread programmed cell death [116]. According to Reape and McCabe [117], senescence and PCD pathways may not overlap, with transcription of PCD genes suppressed until senescence is completed. If this is the case it is possible that cells of ageing seeds may undergo senescence prior to PCD, which could explain the similarity in expression profiles between senescing cells of *Arabidopsis thaliana* [78], [79] and ageing pea seeds, and also the changes in expression patterns as ageing progresses. At the early stages of ageing (8 and 12 d) the expression of a number of PCD-related genes were altered, which was predicted to have an overall effect of repressing PCD. For example, the up-regulation of *thioredoxin-h*, *hexokinase* and *sucrose synthase*, which are negative regulators of PCD, and the down-regulation of *legumain*, *cytochrome c*, *VDAC* and *ANT*, which are all involved in mitochondrial cytochrome c release and the caspase cascade. Notably, *BI-1* was induced at 12 d of ageing, implying a role in repressing PCD in response to ageing for longer than 8 d, but expression subsequently declined to allow PCD to proceed after 15 d of ageing. In accordance with this, several genes associated with PCD, including *DDI1-like*, *NIMA-related kinase*, *aconitase 1* and *cyclophilin D*, were up-regulated only after 15 d of ageing suggesting a wider activation of PCD pathways at this point. Furthermore, repression of oxidative stress-related genes, most of which have antioxidant functions, was enhanced at the later stages of ageing consistent with the view that a decline in antioxidant capacity is required for the progression of PCD [59], [60]. A proposed model of the ageing-induced PCD pathway in seeds is shown in Figure 7. This is a working model based on the model proposed by Kranner *et al*. [4], and whilst evidence is accumulating for parts of the model other parts are yet to be confirmed. Kranner *et al*. [4] also suggested that the mitochondrial pathway involving caspase-like protein activation by cytochrome c could be by-passed via direct activation of caspase-like proteins through thiol nano-switches. The absence of significant changes in *E*_GSSG/2GSH_ until after 25 d of ageing, suggests that this mechanism is unlikely to be involved in the initiation of PCD during the very early stages of ageing prior to viability loss, but could play a role in the later stages. Our updated model identifies PCD-related genes that are differentially expressed during seed ageing and indicates the potential involvement of the ubiquitin-proteasome and ER stress in ageing-induced PCD.

The data reported here represents the average response of all tissues within a population of seeds. Individual seeds will vary in their inherent resistance to ageing, for example antioxidant capacity. Aged samples will comprise live and dead seeds, and there will be some dead cells even in highly viable seed samples. This must be considered when interpreting the results, and may provide some explanation for the lack of a distinct ageing response in terms of processes that are up- or down-regulated. Advances in non-destructive techniques for diagnosing seed viability may in the future provide a means of sorting aged seed populations into live and dead seeds, to allow the finer details of ageing to be studied [118]. In addition, techniques such as laser micro-dissection show promise for enabling tissue specific analyses, which will prove invaluable in understanding processes such as ageing in different seed tissues [119].

This study provides a comprehensive analysis of the relationship between the cellular redox state and global gene expression during seed ageing. The existence of a regulated cell death process is demonstrated by the observation of DNA laddering and transcriptional modulation of genes associated with programmed cell death. Studies of sunflower seed ageing showed that cell death occurred in a synchronous manner, in which all cells simultaneously underwent PCD [120]. It was proposed that ageing induces PCD in seeds once cellular damage has exceeded the capacity for repair. Seed MC and storage temperature are key determinants of the rate of ageing, and probably also of the mechanisms leading to seed viability loss. For example, in rye seeds stored at 40°C with 10% MC nucleic acid degradation resulted in randomly sized DNA fragments, whilst at 14% MC DNA fragments of around 160 base pairs, characteristic of inter-nucleosomal cleavage associated with PCD, were observed [121]. Long term studies to dissect the ageing process at lower temperatures and RH, such as the conditions used for *ex situ* seed storage (e.g. −20°C and 15% RH) may enable the elucidation of molecular markers that could be used to diagnose seed deterioration prior to viability loss, which would be a valuable tool for seed conservation and agriculture. In addition, large scale investigations which combine transcriptomic, proteomic and metabolomic data are necessary to provide the missing pieces in the seed ageing puzzle.

## Supporting Information

Figure S1 **Loss of RNA integrity induced by ageing of pea seeds.** Electropherograms of total RNA samples from seeds of selected ageing treatments: (A) non-aged (0 days); (B) 12 days; (C) 25 days. RNA integrity number (RIN) values were determined using Agilent 2100 Expert software. RNA quality was considered good if the electropherogram showed two distinct peaks for the 25S and 18S bands and a flat baseline. When two peaks were still visible, but the baseline was elevated, RNA was considered partially degraded. RNA was deemed strongly degraded when the two peaks disappeared. Ten categories were defined ranging from 1 (totally degraded RNA) to 10 (intact RNA). Parameters are explained in (A): region A represents low molecular weight RNA; the presence of peaks or smearing in this region indicates the extent of RNA degradation. Peak B and Peak D represent 18S and 25S ribosomal RNA, respectively. The presence of peaks in region C between the 18S and 25S peaks indicate degradation of 25S ribosomal RNA. High molecular weight RNA would appear in region E; peaks or smearing in this region may also result from genomic DNA contamination. Gel-like images of RNA are shown on the far right, with distinct lines for 25S and 18S RNA in (A).
(TIF)

Figure S2 **Hierarchical cluster analysis of 717 ageing-responsive genes.** Genes that were differentially expressed (≥2-fold change in expression compared to non-aged controls) at one or more time points (8 d, 12 d and 15 d) during ageing of *Pisum sativum* seeds. Genes (rows) and experiments (columns) were clustered with The Institute for Genomic Research (TIGR) Multi-experiment Viewer software using Euclidean distance and complete linkage [27].
(TIFF)

Figure S3 **Correlation analysis of gene expression profiles at three time points during ageing of** ***Pisum sativum*** **seeds (0 d vs 8 d, 0 d vs 12 d and 0 d vs 15 d).**
(TIFF)

Figure S4 **Experimental work flow for the gene expression studies using qRT-PCR.** Seed ageing causes RNA degradation, so standard procedures for qRT-PCR could not be followed and a new approach was developed for the assessment of gene expression patterns. Prior to RT, equal amounts of total RNA were spiked with a defined amount of human RNA as an internal standard. Gene expression levels of the human *PBGD* gene were used to assess cDNA synthesis efficiency and to enable normalization of target pea genes.
(TIFF)

Figure S5 **Non-normalised expression of selected genes during seed ageing determined using qRT-PCR.** (a) Distribution of non-normalised expression values of all samples for glucose-6-phosphate dehydrogenase (*G6PDH*), glutathione reductase (*GR*), voltage-dependent anion channel (*VDAC*), adenine nucleotide translocator (*ANT*), β-Tubulin 3 (*Tub*), Actin 1 (*Act*) and elongation Factor-1α (*eF*) as determined by qRT-PCR analysis. (b) to (i) Non-normalised expression values during ageing. Data points are means (n = 5). Solid lines represent linear regression and dotted curves indicate 95% confidence intervals. Asterisks denote genes with a regression slope deviating significantly from zero (linear regression analysis, *P*<0.001). Correlation coefficient (R^2^) of regression analysis: *PBGD* = 0.3801; *G6PDH* = 0.04478; *GR* = 0.9777; *VDAC* = 0.8397; *ANT* = 0.9619; *Tub* = 0.85; *Act* = 0.8619; *eF* = 0.8756.
(TIFF)

Table S1 **Primer sequences used for qRT-PCR analysis of β-tubulin-3 (** ***Tub*** **), elongation factor-1α (** ***eF*** **), actin 1 (** ***Act*** **), glutathione reductase (** ***GR*** **), glucose-6-phosphate dehydrogenase (** ***G6PDH*** **), voltage-dependent anion-selective channel (** ***VDAC*** **) and an adenine nucleotide translocator (** ***ANT*** **) gene expression.**
(DOCX)

Table S2 **Genes showing altered expression during the artificial ageing of pea seeds.** Expression values correspond to the log transformed ratio of expression between aged (8, 12 and 15 d) and non-aged (0 d) seed. Genes representing at least a 2-fold change (log_2_ ratio ≥1 for up-regulation and ≤−1 for down-regulation) at one or more time points are included. Genes which showed a change in expression corresponding to a log_2_ ratio ≥1.0 or ≤−1.0 are shaded in light magenta (up-regulation) or light green (down-regulation), and those which were changed by a log_2_ ratio of ≥1.5 or ≤−1.5 are shaded in dark magenta (up-regulation) or dark green (down-regulation). Genes that were assigned a KOG annotation are arranged in KOG functional categories.
(XLS)

Table S3 **Hierarchical clustering of ageing-responsive genes.** All 717 differentially expressed genes were grouped into 16 clusters according to expression profiles during the ageing time course. Genes representing at least a 2-fold change (log_2_ ratio ≥1.0 or ≤−1.0) at one or more time points are included. Expression values corresponding to a log_2_ ratio ≥1.0 or ≤−1.0 are shaded in light magenta (up-regulation) or light green (down-regulation), and those which were changed by a log_2_ ratio of ≥1.5 or ≤−1.5 are shaded in dark magenta (up-regulation) or dark green (down-regulation). For KOG annotated genes, the functional category ID and description are shown.
(XLS)

Table S4 **Functional classification of ageing-responsive genes related to PCD, oxidative stress, ubiquitin-proteolysis and signaling etc.** Genes representing at least a 2-fold change (log_2_ ratio ≥1.0 or ≤−1.0) at one or more time points are included. Expression values corresponding to a log_2_ ratio ≥1.0 or ≤−1.0 are shaded in light magenta (up-regulation) or light green (down-regulation), and those which were changed by a log_2_ ratio of ≥1.5 or ≤−1.5 are shaded in dark magenta (up-regulation) or dark green (down-regulation). For KOG annotated genes, the functional category ID and description are shown, and for all genes the cluster to which each gene belongs is also shown.
(XLS)
