# Correction: Transcriptome-Wide Mapping of Pea Seed Ageing Reveals a Pivotal Role for Genes Related to Oxidative Stress and Programmed Cell Death

**DOI:** 10.1371/annotation/f8467b75-4eef-4c44-ad20-eee34c784a66

**Published:** 2014-01-06

**Authors:** Hongying Chen, Daniel Osuna, Louise Colville, Oscar Lorenzo, Kai Graeber, Helge Küster, Gerhard Leubner-Metzger, Ilse Kranner

The fifth author was incorrectly indicated as being affiliated with institution number 5. Kai Graeber was only affiliated with institution number 4, Institute for Biology II, Botany/Plant Physiology, Faculty of Biology, Albert-Ludwigs-University Freiburg, Freiburg, Germany, at the time of the article's publication.

In addition, the Current Address of the fifth author was incorrectly omitted in the article byline. The Current Address of the fifth author is: School of Biological Sciences, Plant Molecular Science and Centre for Systems and Synthetic Biology, Royal Holloway, University of London, Egham, Surrey, United Kingdom. 

