# Supplementary figures and images for: Transcriptome-Wide Mapping of Pea Seed Ageing Reveals a Pivotal Role for Genes Related to Oxidative Stress and Programmed Cell Death
Source: PLoS One. 2013 Oct 29;8(10):e78471. doi: 10.1371/journal.pone.0078471 (PMC3812160; doi:10.1371/journal.pone.0078471)

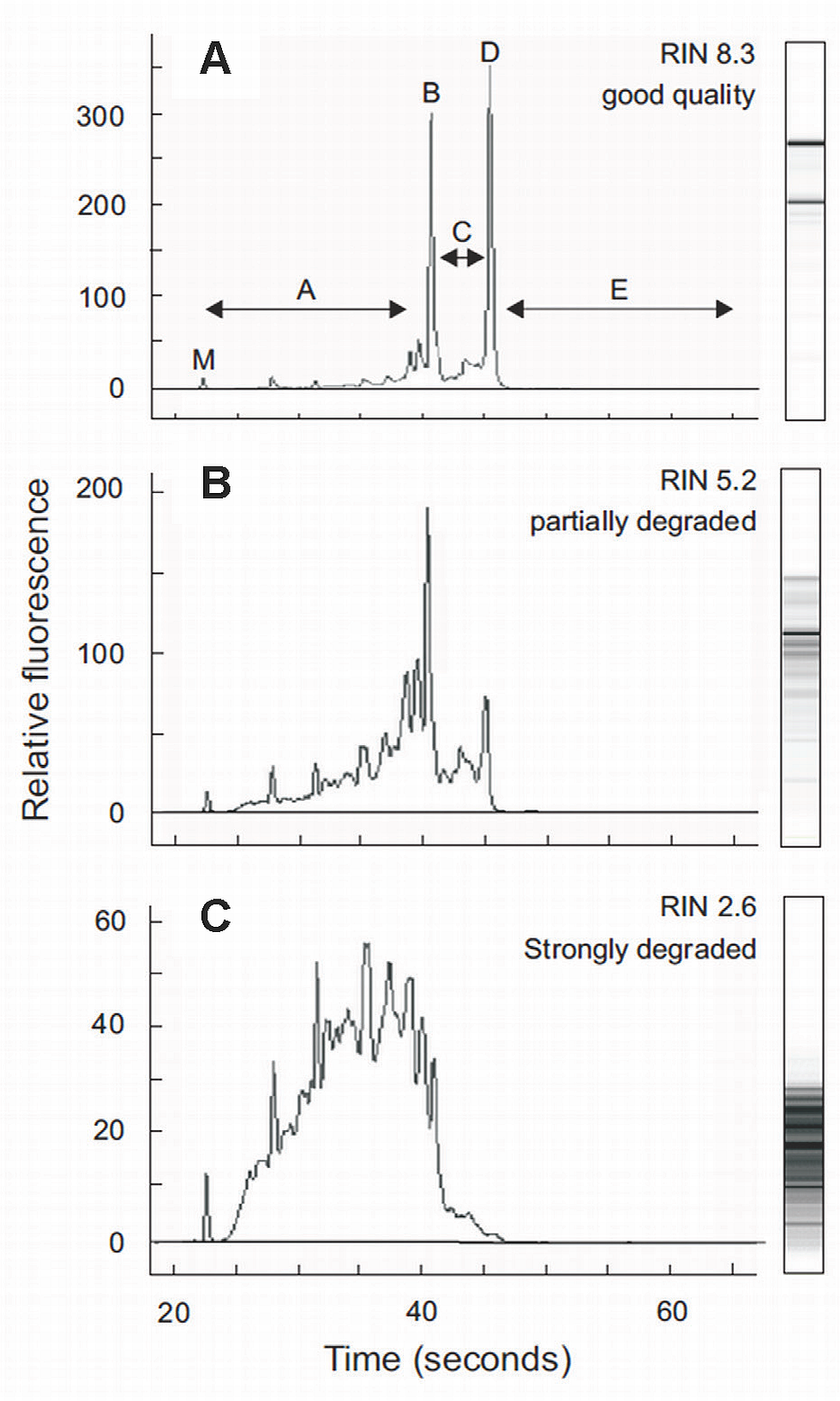

Supplement: Figure S1 — Loss of RNA integrity induced by ageing of pea seeds. Electropherograms of total RNA samples from seeds of selected ageing treatments: (A) non-aged (0 days); (B) 12 days; (C) 25 days. RNA integrity number (RIN) values were determined using Agilent 2100 Expert software. RNA quality was considered good if the electropherogram showed two distinct peaks for the 25S and 18S bands and a flat baseline. When two peaks were still visible, but the baseline was elevated, RNA was considered partially degraded. RNA was deemed strongly degraded when the two peaks disappeared. Ten categories were defined ranging from 1 (totally degraded RNA) to 10 (intact RNA). Parameters are explained in (A): region A represents low molecular weight RNA; the presence of peaks or smearing in this region indicates the extent of RNA degradation. Peak B and Peak D represent 18S and 25S ribosomal RNA, respectively. The presence of peaks in region C between the 18S and 25S peaks indicate degradation of 25S ribosomal RNA. High molecular weight RNA would appear in region E; peaks or smearing in this region may also result from genomic DNA contamination. Gel-like images of RNA are shown on the far right, with distinct lines for 25S and 18S RNA in (A). (TIF) [file pone.0078471.s001.tif]

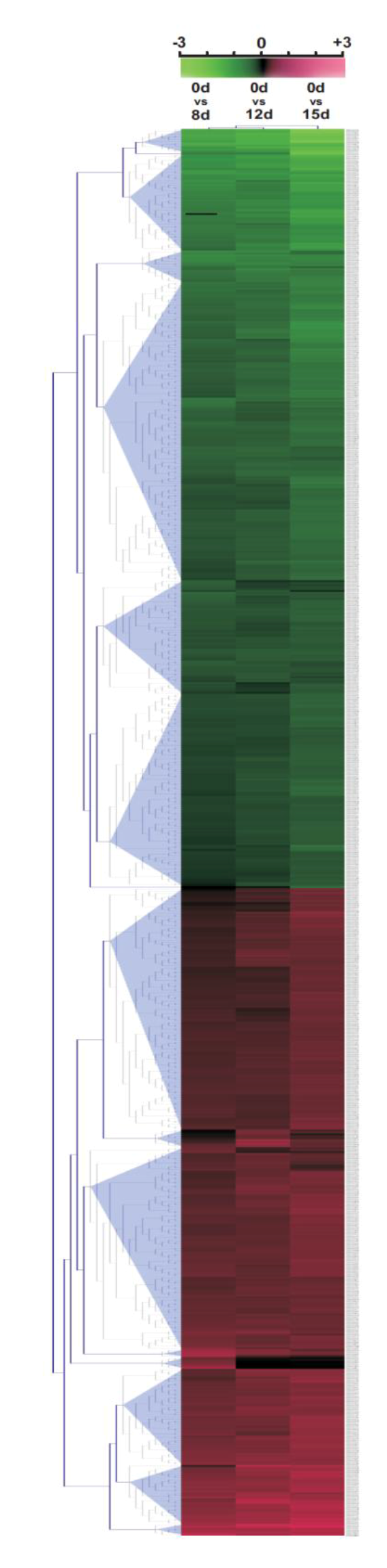

Supplement: Figure S2 — Hierarchical cluster analysis of 717 ageing-responsive genes. Genes that were differentially expressed (≥2-fold change in expression compared to non-aged controls) at one or more time points (8 d, 12 d and 15 d) during ageing of Pisum sativum seeds. Genes (rows) and experiments (columns) were clustered with The Institute for Genomic Research (TIGR) Multi-experiment Viewer software using Euclidean distance and complete linkage [27]. (TIFF) [file pone.0078471.s002.tif]

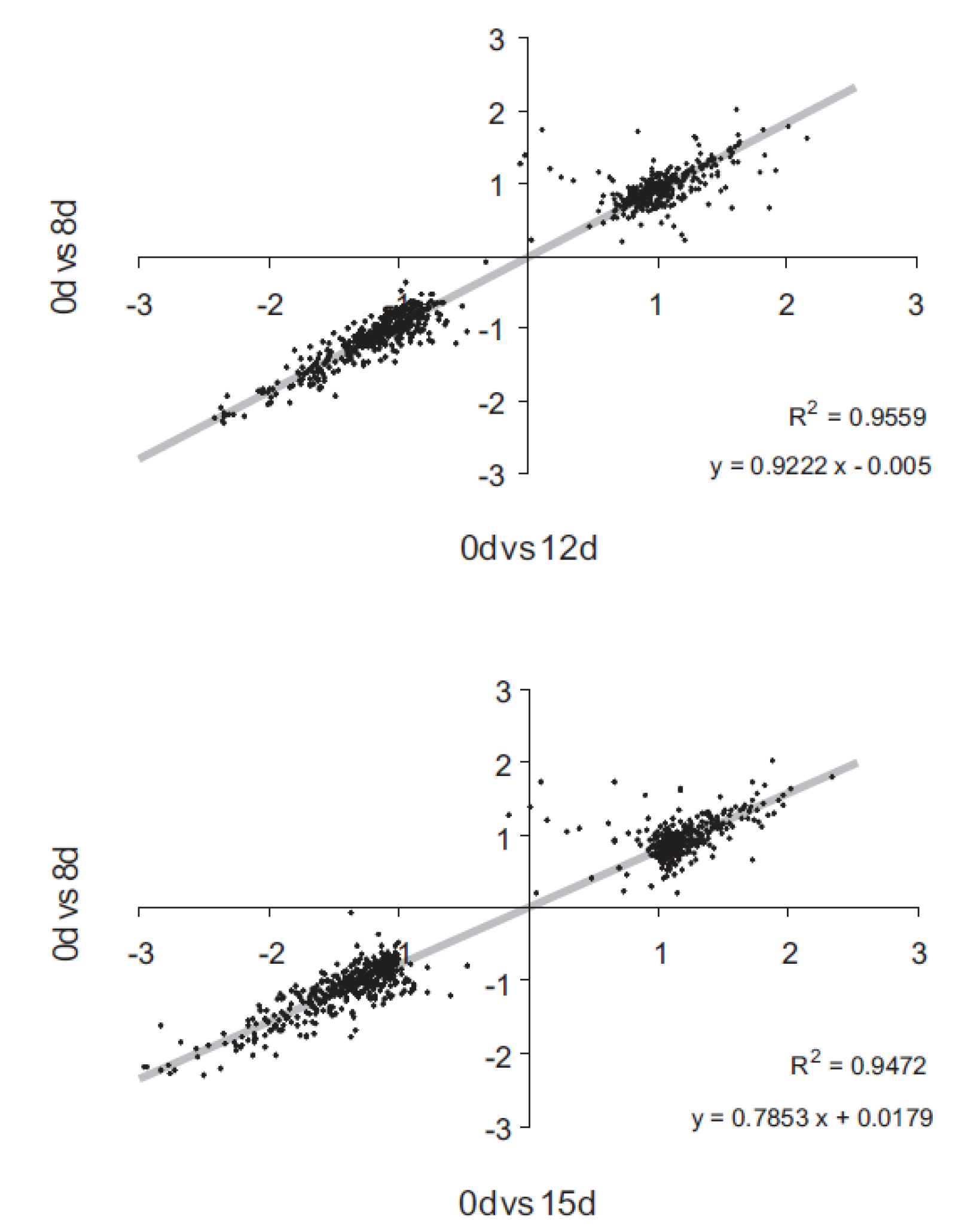

Supplement: Figure S3 — Correlation analysis of gene expression profiles at three time points during ageing of Pisum sativum seeds (0 d vs 8 d, 0 d vs 12 d and 0 d vs 15 d). (TIFF) [file pone.0078471.s003.tif]

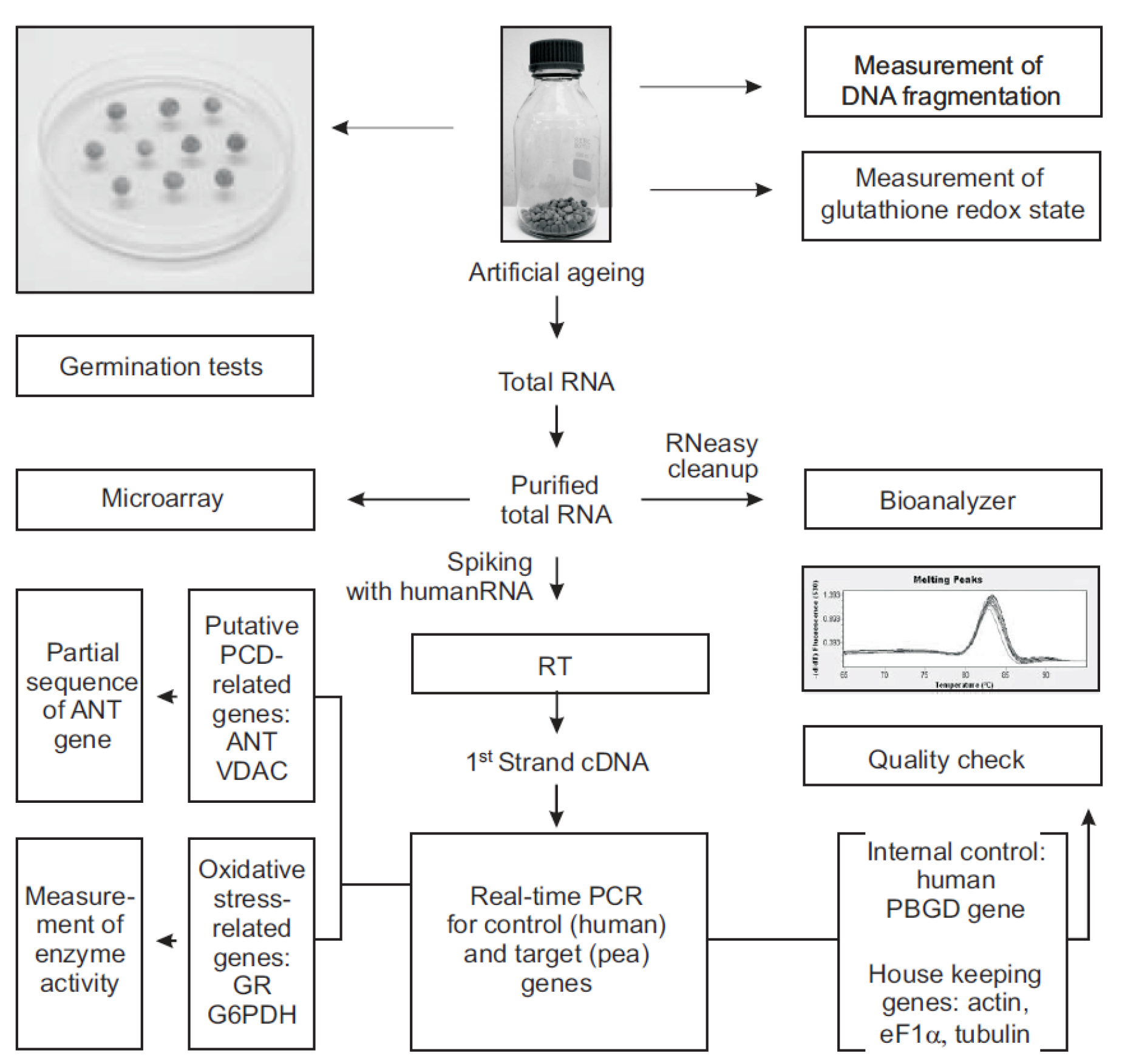

Supplement: Figure S4 — Experimental work flow for the gene expression studies using qRT-PCR. Seed ageing causes RNA degradation, so standard procedures for qRT-PCR could not be followed and a new approach was developed for the assessment of gene expression patterns. Prior to RT, equal amounts of total RNA were spiked with a defined amount of human RNA as an internal standard. Gene expression levels of the human PBGD gene were used to assess cDNA synthesis efficiency and to enable normalization of target pea genes. (TIFF) [file pone.0078471.s004.tif]

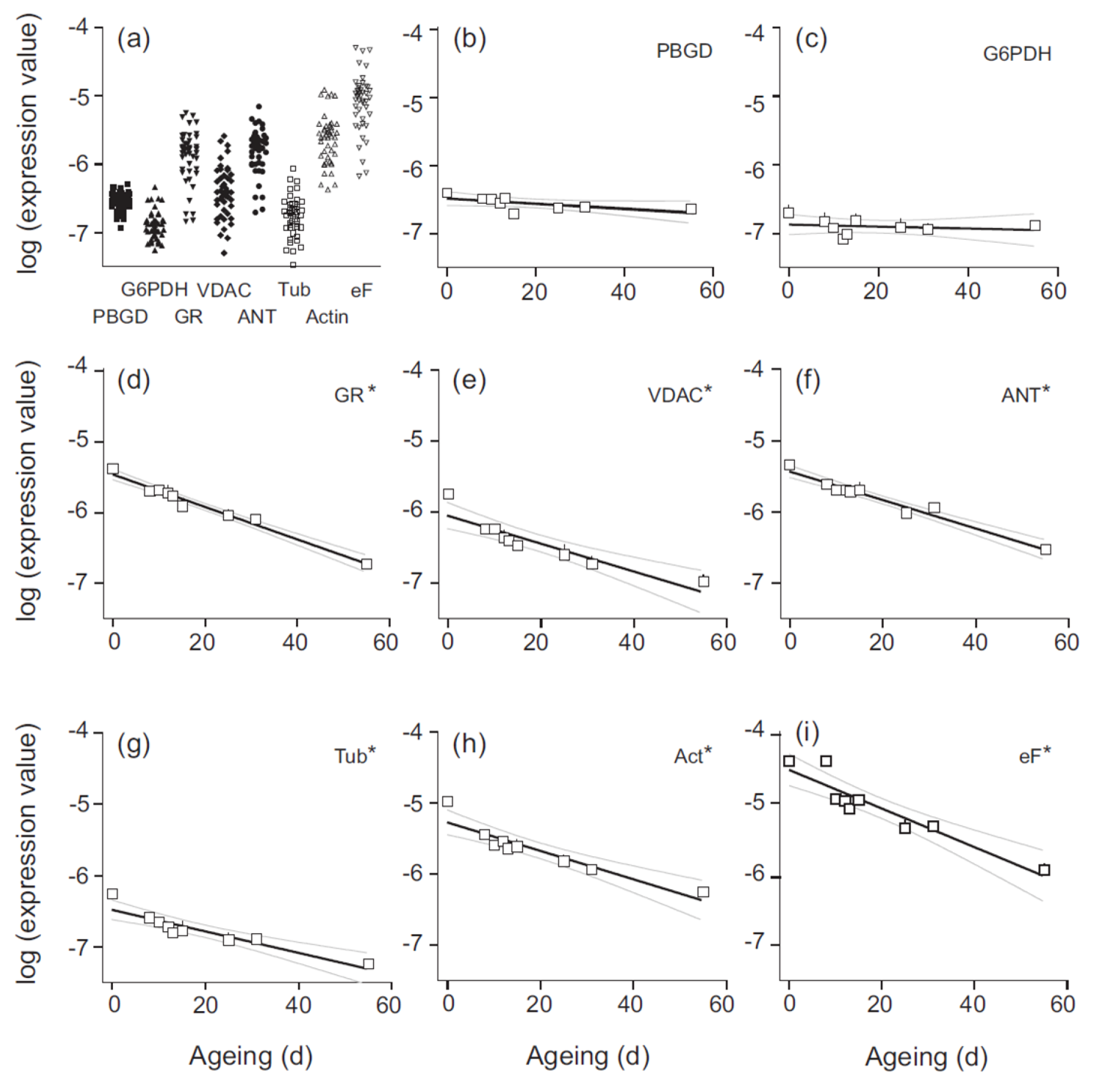

Supplement: Figure S5 — Non-normalised expression of selected genes during seed ageing determined using qRT-PCR. (a) Distribution of non-normalised expression values of all samples for glucose-6-phosphate dehydrogenase (G6PDH), glutathione reductase (GR), voltage-dependent anion channel (VDAC), adenine nucleotide translocator (ANT), β-Tubulin 3 (Tub), Actin 1 (Act) and elongation Factor-1α (eF) as determined by qRT-PCR analysis. (b) to (i) Non-normalised expression values during ageing. Data points are means (n = 5). Solid lines represent linear regression and dotted curves indicate 95% confidence intervals. Asterisks denote genes with a regression slope deviating significantly from zero (linear regression analysis, P<0.001). Correlation coefficient (R2) of regression analysis: PBGD = 0.3801; G6PDH = 0.04478; GR = 0.9777; VDAC = 0.8397; ANT = 0.9619; Tub = 0.85; Act = 0.8619; eF = 0.8756. (TIFF) [file pone.0078471.s005.tif]
