# Supplementary material for: Transcriptome-Wide Mapping of Pea Seed Ageing Reveals a Pivotal Role for Genes Related to Oxidative Stress and Programmed Cell Death
Source: PLoS One. 2013 Oct 29;8(10):e78471. doi: 10.1371/journal.pone.0078471 (PMC3812160; doi:10.1371/journal.pone.0078471)
Supplement: Table S1 — Primer sequences used for qRT-PCR analysis of β-tubulin-3 ( Tub ), elongation factor-1α ( eF ), actin 1 ( Act ), glutathione reductase ( GR ), glucose-6-phosphate dehydrogenase ( G6PDH ), voltage-dependent anion-selective channel ( VDAC ) and an adenine nucleotide translocator ( ANT ) gene expression. (DOCX) [file pone.0078471.s006.docx]

**Supporting Table S1**

| **Name** | **Forward primer sequence (5’-3’)** | **Reverse primer sequence (5’-3’)** | **Length (bp)** |
| --- | --- | --- | --- |
| *Tub* | GGCCGCTTCGTTCCCAGAG | GCCCAGTTATTCCCAGCACCAC | 137 |
| *eF* | CCAGTTCTTGATTGCCACACCTCT | TAACCATACCGGCATCACCATTCT | 133 |
| *Act* | CAACCCAAAGGCCAACAGAGAAA | CCACTTGCATAGAGGGAGAGGACA | 108 |
| *GR* | GGTGAGGAGTTGATTGCGGATGT | GCTAGGGATGTTGGTGCGTGAAT | 147 |
| *G6PDH* | AGATTTTCACGCCGCTTTTACAC | AGCTTCTGCGGGACCTCTACTTC | 89 |
| *VDAC* | ACATCTGGGAAGGTGGAACTACAA | ATCAGCACCAAAAGCAAGAGCAT | 132 |
| *ANT* | GTTTGCACCGGCACCTTTGAA | CCCATTGACACCGTGCGTAGAA | 135 |

**Supporting Table S1.** Primer sequences used for qRT-PCR analysis of β-tubulin-3 (*Tub*), elongation factor-1α (*eF*), actin 1 (*Act*), glutathione reductase (*GR*), glucose-6-phosphate dehydrogenase (*G6PDH*), voltage-dependent anion-selective channel (*VDAC*) and an adenine nucleotide translocator (*ANT*) gene expression.
